# Global Transcriptomic and Characteristics Comparisons between Mouse Fetal Liver and Bone Marrow Definitive Erythropoiesis

**DOI:** 10.3390/cells13131149

**Published:** 2024-07-05

**Authors:** Chengjie Gao, Huan Zhang, Yaomei Wang, Shihui Wang, Xinhua Guo, Yongshuai Han, Huizhi Zhao, Xiuli An

**Affiliations:** 1 School of Life Sciences, Zhengzhou University, Zhengzhou 450001, China; chengjie_gao@163.com (C.G.);; 2 Laboratory of Membrane Biology, New York Blood Center, New York, NY 10065, USA; 3 Department of Hematology, The Affiliated Cancer Hospital of Zhengzhou University & Henan Cancer Hospital, Zhengzhou 450008, China; 4 Institute of Hematology, People’s Hospital of Zhengzhou University, Zhengzhou 450003, China

**Keywords:** definitive erythropoiesis, transcriptome analysis, cell cycle, bone marrow, fetal liver

## Abstract

Erythropoiesis occurs first in the yolk sac as a transit “primitive” form, then is gradually replaced by the “definitive” form in the fetal liver (FL) during fetal development and in the bone marrow (BM) postnatally. While it is well known that differences exist between primitive and definitive erythropoiesis, the similarities and differences between FL and BM definitive erythropoiesis have not been studied. Here we performed comprehensive comparisons of erythroid progenitors and precursors at all maturational stages sorted from E16.5 FL and adult BM. We found that FL cells at all maturational stages were larger than their BM counterparts. We further found that FL BFU-E cells divided at a faster rate and underwent more cell divisions than BM BFU-E. Transcriptome comparison revealed that genes with increased expression in FL BFU-Es were enriched in cell division. Interestingly, the expression levels of glucocorticoid receptor *Nr3c1*, *Myc* and *Myc* downstream target *Ccna2* were significantly higher in FL BFU-Es, indicating the role of the Nr3c1-Myc-Ccna2 axis in the enhanced proliferation/cell division of FL BFU-E cells. At the CFU-E stage, the expression of genes associated with hemoglobin biosynthesis were much higher in FL CFU-Es, indicating more hemoglobin production. During terminal erythropoiesis, overall temporal patterns in gene expression were conserved between the FL and BM. While biological processes related to translation, the tricarboxylic acid cycle and hypoxia response were upregulated in FL erythroblasts, those related to antiviral signal pathway were upregulated in BM erythroblasts. Our findings uncovered previously unrecognized differences between FL and BM definitive erythropoiesis and provide novel insights into erythropoiesis.

## 1. Introduction

Erythropoiesis is a process by which red blood cells are produced. It starts at the yolk sac as early as embryonic day 7.5 (E7.5) in mice [1,2,3] and 3 weeks in humans [2,4]. This early transit wave of erythropoiesis is referred to as primitive erythropoiesis, during which large nucleated erythroid cells are produced from hemangioblasts [3,4,5]. The second wave consists of a transient wave of definitive erythroid progenitors that emerge from the yolk sac at E8.25–8.5 and seed the fetal liver (FL) where they generate definitive erythroid precursors that enucleate to become the first circulating RBCs in the fetus [5]. The third wave of erythropoiesis produces erythroid cells from hematopoietic stem cells (HSCs) that seed and differentiate within the fetal liver by ~E14.5 in the mouse [1,2,3] and 7–8 weeks in the human embryo [2,3,4]. After birth, the definitive erythropoiesis occurs in the bone marrow (BM) that persists throughout a lifetime.

In contrast to dramatic differences between primitive and definitive erythropoiesis [6,7], FL and BM definitive erythropoiesis share many phenotypic similarities. They undergo the same maturational process during which HSCs differentiate into the lineage-committed erythroid progenitor, burst colony forming unit-erythroids (BFU-Es), which further differentiate into the late-stage erythroid progenitor, colony forming unit-erythroids (CFU-Es). CFU-Es undergo terminal erythroid differentiation to sequentially become proerythroblasts (Pros), basophilic erythroblasts (Basos), polychromatic erythroblasts (Polys) and orthochromatic erythroblasts (Orthos). Orthos expel out their nuclei to generate reticulocytes. Finally, reticulocytes mature into red blood cells (RBCs) in circulation. We have documented that erythroid cells at each distinct maturational stage in both the FL and BM can be distinguished using the same surface markers [8]. BFU-Es and CFU-Es can be isolated based on the expression levels of CD71 on the Lin^−^Kit^+^ cells [8,9]. Pros, Basos, Polys and Orthos of both the FL and BM can be separated using Ter119, CD44 and forward scatter (FSC) as parameters [8,10]. Despite the phenotypic similarities described above, functional and molecular differences between FL and BM definitive erythropoiesis are expected due to the rapid growth of the fetus during fetal development, while BM erythropoiesis occurs at a steady pace in adult mice. In addition, the microenvironments for FL and BM definitive erythropoiesis are different. However, the extent of similarities and differences between FL and BM definitive erythropoiesis have not been characterized. In the present study, we performed comprehensive comparisons of erythroid progenitors and precursors at all maturational stages sorted from both E16.5 FL and adult BM. We have uncovered previously unknown differences between FL and BM definitive erythropoiesis, including cell size and BFU-E proliferation/cell cycle. Transcriptome comparisons revealed differences in the expression of genes involved in the cell cycle, hemoglobin biosynthesis, immunomodulation, translation, the tricarboxylic acid (TCA) cycle and response to hypoxia and viruses. Our findings provide novel insights into erythropoiesis and our transcriptomes of erythroid progenitors and precursors at all maturational stages provide rich resources for future mechanistic studies.

## 2. Materials and Methods

### 2.1. Reagents and Mice

All the reagents including antibodies, primers and chemicals used in the present study are listed in Table S1. C57BL/6 mice were purchased from Jackson Lab (Bar Harbor, ME, USA). The mice were maintained at the New York Blood Center Animal Facility. 12-week-old male and female mice were used for the study. Animal protocols were reviewed and approved by the Institutional Animal Care and Use Committee of New York Blood Center.

### 2.2. Fluorescence-Activated Cell Sorting

Single cell suspensions of E16.5 FL and adult BM were prepared as previously described [10,11]. To sort erythroid progenitors, lineage^-^ cells were enriched by depletion of lineage^+^ cells and the lineage^-^ cells were then stained as previously described [8]. Cells at a concentration of 10^8^/mL were blocked with rat anti-mouse CD16/CD32 antibodies and then stained with biotin-conjugated anti mouse lineage antibodies including CD11b, Gr1, CD3e, CD45R/B220 and Ter-119. Subsequently, lineage^-^ cells were enriched by Anti-Biotin Microbeads (Miltenyi Biotec, Auburn, CA, USA) according to the instructions from the manufacture. Next, the lineage- cells at a concentration of 10^7^/mL were blocked with rat anti-mouse CD16/CD32 antibodies and then stained with BV421-conjugated anti-mouse CD16/32, FITC-conjugated anti-mouse CD71, APC-conjugated anti-mouse CD34, APC-conjugated anti-mouse Ly-6A/E (Sca-1), PerCP-conjugated Streptavidin, APC-Cyanine7 conjugated anti-mouse CD117 (c-kit) and eFluor450-conjugated anti-mouse CD41a. For erythroid precursors, single cells at a concentration of 10^7^/mL were blocked with rat anti-mouse CD16/CD32 antibodies and then stained with BV421-conjugated anti-mouse Ter-119, APC-conjugated anti-mouse CD44, APC-Cy7-conjugated anti-mouse CD45, APC-Cy 7–conjugated anti-mouse CD11b and APC-Cy7-conjugated anti-mouse Gr1. 7-aminoactinomycin D (7AAD) was used as viability marker. Erythroid progenitors and precursors were sorted on a BD FACSAria™ Fusion cell sorter (BD, Franklin Lakes, NJ, USA) according to the gating strategy described previously [8,12].

### 2.3. Cytospin Preparation and MGG (May–Grunwald–Giemsa) Staining

Cytospins were prepared on coated slides with ~1 × 10^4^ cells by using the Thermo Scientific Shandon 4 Cytospin (Thermo Fisher Scientific, Waltham, MA, USA). For MGG staining, the slides were stained with May–Grunwald (Sigma MG500, Kawasaki-shi, Japan) solution for 5 min and then rinsed for 90 s with 40 mM Tris-buffer (pH 7.4). Subsequently, the slides were stained with Giemsa solution (Sigma GS500, Kawasaki-shi, Japan) diluted 10 times with water for 15 min. Finally, the slides were rinsed twice with water. The cells were imaged using a Leica DM2000 inverted microscope (Leica Microsystems, Buffalo Grove, IL, USA).

### 2.4. Single Cell Culture of Erythroid Progenitors In Vitro

Single BFU-E or CFU-E cells were sorted to a 96-well plate and cultured with StemSpan™ SFEM medium (STEMCELL Technologies, Vancouver, BC, Canada) containing 100 ng/mL recombinant murine stem cell factor (rmSCF, STEMCELL Technologies, Vancouver, BC, Canada), 40 ng/mL recombinant murine insulin-like growth factor-1 (rmIGF-1, STEMCELL Technologies, Vancouver, BC, Canada), 2 U/mL recombinant human erythropoietin (rhEPO, Gibco, Billings, MT, USA), 100 U/mL penicillin and 100 μg/mL streptomycin and incubated at 37 °C in a humidified atmosphere (relative humidity > 80%) with 5% CO_2_.

### 2.5. Cell Division Tracing Assay

Sorted BM and FL erythroid progenitors were stained with CellTrace™ Violet dye (Thermo Fisher Scientific, Eugene, OR, USA) according to the instructions from the manufacturer and cultured in the medium as described above. The fluorescence intensity of the dye was detected by flow cytometry at different time points. Cell division numbers were calculated based on the standard that the fluorescence intensity of offspring is reduced by half compared to that of the parents.

### 2.6. RNA-Seq and Real-Time PCR Analyses

RNA was purified from the sorted erythroid cells as described previously [8,10,12]. The integrity of RNA samples was detected by Agilent 2100 RNA nano assay kit (Agilent Technologies, Santa Clara, CA, USA). Samples with an RNA integrity number of >9 were used for construction of DNA library. For erythroid progenitors BFU-E and CFU-E, the library was constructed by low-input Clontech SMART-Seq HT with Nxt HT (Takara Bio USA, San Jose, CA, USA), and then sequenced by an Illumina Nova Seq 6000 (Illumina, San Diego, CA, USA) using 50 bp paired-end strategy. For erythroblasts, the library was constructed by Illumina VAHTS Universal V6 RNA-seq Library Prepkit NR 604-01/02 (Illumina, San Diego, CA, USA), and then sequenced by an Illumina NovaSeq 6000 (Illumina, San Diego, CA, USA) using 150 bp paired-end strategy. Low-quality reads were removed and filtered by fastp v0.23.4 [13]. Filtered reads were quantified by Kallisto v0.48.0 [14] using the mm10 transcriptome index. Pairwise comparison of two groups was performed by DESeq2 v1.44.0 [15]. The cutoffs of a fold change of ≥2, adjusted *p* of ≤0.05 and ≥5 transcript per million (TPM) at least in one group were adopted to identify DEGs in the pairwise comparisons. Principal component analysis (PCA) was conducted on log-transformed normalized counts in gene expression analysis. Heatmap was conducted on log-transformed normalized counts in gene expression analysis. Gene ontology (GO) and pathway enrichment analyses were applied by Metascape v3.5 [16]. GO terms and pathways with a q-value of <0.001 were considered significant, and the top 5 significant terms with the smallest q-values were listed in the results. Clustering analyses were done by k-means on log-transformed normalized counts in transcriptome analysis.

### 2.7. In Vivo 5-Ethynyl-2′-Deoxyuridine Incorporation Assay

5-ethynyl-2′-deoxyuridine (Edu) (500 μg per mouse) were injected into mice by i.p. 30 min before sacrifice. Lineage^−^ cells were enriched and stained with fluorescent antibodies for erythroid progenitor analysis, and then stained using EdU according to the manufacturer’s instructions (Invitrogen, Waltham, MA, USA). 7AAD was used as a DNA dye. Cells were analyzed by using BD FACSDiva Version 6.1.2 software on a LSR Fortessa flow cytometer (Becton Dickinson, East Rutherford, NJ, USA).

### 2.8. Statistics

FlowJo™ v10 software was used to analyze flow cytometric data. ImageJ software v1.54j was used to quantify the cell area of the sorted erythroid progenitors and precursors. GraphPad Prism 8 software was used for statistical analysis. All data were reported as mean ± SEM. Differences between two groups were calculated by unpaired student’s *t* test.

## 3. Results

### 3.1. FL Definitive Erythroid Progenitors and Precursors at All Maturational Stages Were Larger Than Their BM Counterparts

**Figure 1:**
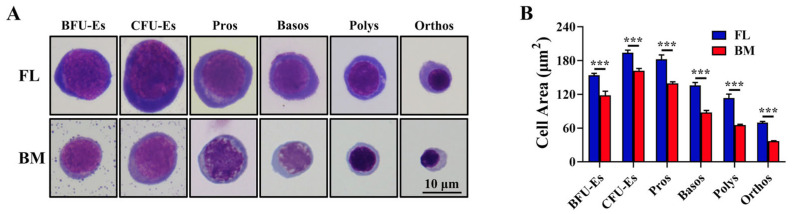
Morphology of mouse FL and BM erythroid cells. (**A**) Representative images of erythroid cells at all maturational stages from mouse E16.5 FL and adult BM. (**B**) Quantitative analysis of cell area of erythroid progenitors and precursors at all maturational stages. In total, 30 cells at each stage were used for quantification. *** *p* < 0.001. FL: fetal liver; BM: bone marrow; BFU-Es: burst colony forming unit-erythroids; CFU-Es: colony forming unit-erythroids; Pros: proerythroblasts; Basos: basophilic erythroblasts; Polys: polychromatic erythroblasts; Orthos: orthochromatic erythroblasts.

To compare the similarities and differences between FL and BM definitive erythropoiesis in a stage-specific manner, we sorted erythroid progenitors and precursors at all maturational stages using the methods we and others have developed. Specifically, FL BFU-E and CFU-E cells were defined as 10% Lin^−^c-Kit^+^CD71^Low^ and 20% Lin^−^c-Kit^+^CD71^High^, respectively, according to Flygare J et al. [9], and BM BFU-E and CFU-E cells were sorted as Lin^−^Kit^+^CD71^−^ and Lin^−^Kit^+^CD71^High^, respectively, according to Zhang H et al. [8]. Pros, Basos, Polys and Orthos from both FL and BM were sorted using Ter119, CD44 and forward scatter as parameters, as described previously [10,17]. The gating strategy for sorting FL erythroid progenitors, BM erythroid progenitors, FL erythroblasts and BM erythroblasts is shown in Figure S1. Representative images of the sorted cells are shown in Figure 1A, which revealed that FL erythroid cells at all maturational stages were larger than their BM counterparts. The quantification of the cell area is shown in Figure 1B, which revealed that the size of the FL BFU-Es, CFU-Es and Pros was ~1.3-fold larger than their BM counterparts. The differences in cell size increased during terminal erythroid differentiation. The size of FL Basos and Polys was ~1.6-fold larger than their BM counterparts, and the size of FL Orthos was almost 2 times larger than BM Ortho.

### 3.2. Higher Proliferation Capability of FL BFU-E Cells

**Figure 2:**
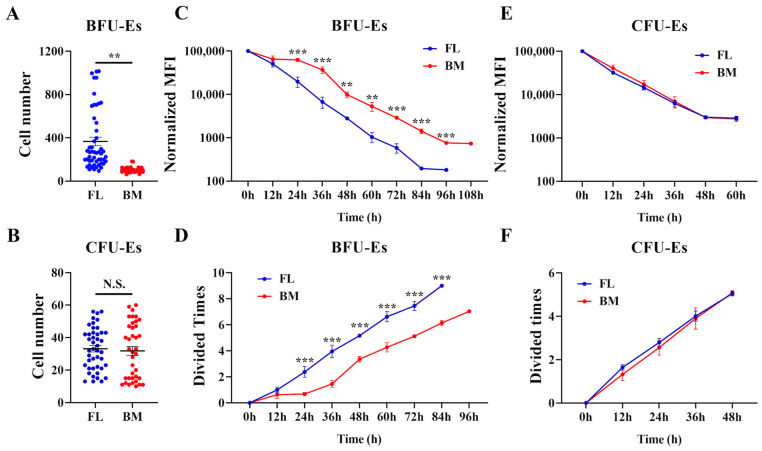
Proliferation capability of mouse FL and BM erythroid cells. (**A**) Numbers of cell grown from one sorted FL or BM BFU-E cell after culturing for 96 h. (**B**) Numbers of cell grown from one sorted FL or BM CFU-E cell after culturing for 48 h. (**C**) Normalized violet MFI of FL or BM BFU-Es detected at indicated time points. N = 3. (**D**) Numbers of cell division of FL or BM BFU-Es calculated based on changes in MFI. N = 3. (**E**) Normalized violet MFI of FL or BM CFU-Es detected at indicated time points. N = 3. (**F**) Numbers of cell division of FL or BM CFU-Es calculated based on changes in MFI. MFI: mean fluorescence intensity N = 3. N.S. Not Significant. ** *p* < 0.01, *** *p* < 0.001.

We next compared the proliferation of FL and BM BFU-E and CFU-E cells using single cell culture. Figure 2A shows that after culturing for 96 h (when the cells stopped dividing [9]), one FL BFU-E cell gave rise to 94–1015 cells, while one BM BFU-E cell only gave rise to 62–180 cells, indicating a higher proliferation capability as well as heterogeneity of FL BFU-E cells compared to BM BFU-E cells. Interestingly, in contrast to the differences between FL and BM BFU-E cells, FL and BM CFU-Es exhibited similar proliferation capability as evidenced by similar numbers of cells given by one CFU-E after culturing for 48 h (Figure 2B). We further monitored cell division using a violet dye. The representative profile of cell division of the sorted FL BFU-E, BM BFU-E, FL CFU-E and BM CFU-E cells is shown in Figure S2A–D, respectively. Quantitative analyses of mean fluorescence intensity (MFI) revealed that FL BFU-E cells divided at a faster rate in the first 36 h as evidenced by the faster decrease in MFI of FL cells compared with BM cells (Figure 2C). Furthermore, the MFI detected at 96 h was identical to that detected at 84 h, indicating the division of FL BFU-E ceased after 84 h (Figure 2C). BM BFU-Es ceased division after 96 h as demonstrated by the same MFI detected at 96 h and 108 h. The calculation of cell division times using changes in MFI showed that while FL BFU-Es divided nine times, BM BFU-Es divided seven times (Figure 2D). For CFU-Es, the division rates of FL CFU-Es and BM CFU-Es were the same (Figure 2E), and on average both divided five times (Figure 2F).

### 3.3. GO Terms Related to Cell Cycle Were Upregulated in FL BFU-Es Than in BM BFU-Es

**Figure 3:**
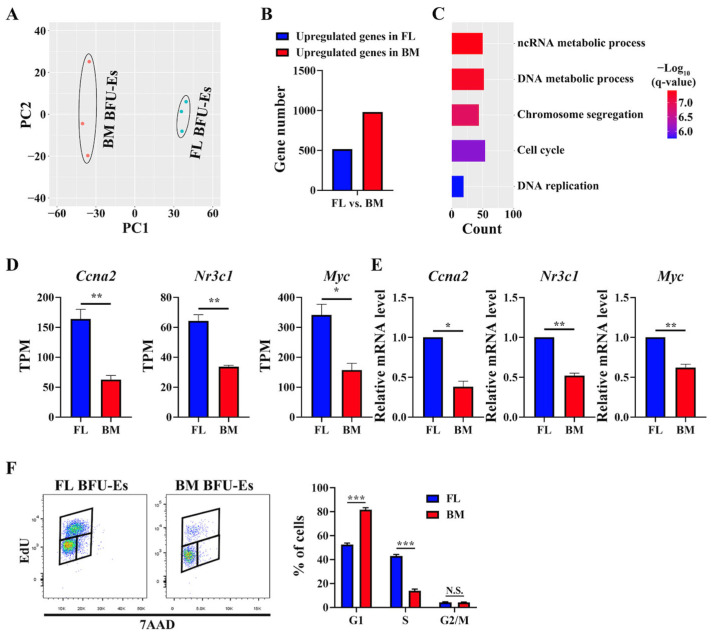
Comparisons between mouse FL and BM BFU-E cells. (**A**) Principal component analyses of transcriptomes showing separation of FL BFU-Es and BM BFU-Es. (**B**) Bar plot of differentially expressed gene (DEG) numbers between FL BFU-Es and BM BFU-Es. (**C**) Bar plot of GO terms enriched in genes with higher expression in FL BFU-Es than BM BFU-Es. (**D**) Expression levels of *Ccna2*, *Nr3c1* and *Myc* in FL and BM BFU-E cells from RNA-seq by TPM. (**E**) Expression levels of *Ccna2*, *Nr3c1* and *Myc* in FL and BM BFU-E cells as assessed by real-time PCR. *Gapdh* was used as control. (**F**) Cell cycle analyses of FL and BM BFU-E cells as assessed by in vivo EdU uptake by flow cytometry. N = 3. N.S.: Not Significant. * *p* < 0.05. ** *p* < 0.01, *** *p* < 0.001. q-value represents adjusted *p*. PC: principal component.

To define the differences between FL and BM BFU-E cells at the molecular level, we compared the transcriptomes of the FL and BM BFU-E cells. Principle component analysis (PCA) showed a clear separation of FL BFU-E and BM BFU-E (Figure 3A). BFU-E cells from both FL and BM expressed around 10,750 genes, among which 992 genes were differentially expressed with 354 genes upregulated and 638 downregulated in FL BFU-Es compared to BM BFU-Es (Figure 3B). The DEGs are listed in Table S2. Analyses of the DEGs revealed that, consistent with the higher proliferation capability of FL BFU-E cells, top GO terms upregulated in FL BFU-Es were related to the cell cycle process (Figure 3C). Specifically, the expression levels of *Ccna2* were significantly higher in FL BFU-Es than in BM BFU-Es (Figure 3D). Additionally, the expression levels of glucocorticoid receptor *Nr3c1* and transcription factor *Myc* were also higher in FL BFU-Es than in BM-BFUEs (Figure 3D). The differences in the expression of these genes were confirmed by real-time PCR (Figure 3E). To examine the cell cycle status of FL and BM BFU-E cells in vivo, we performed the in vivo EdU incorporation assay. As shown in Figure 3F, the percentages of cells in S-phase were much higher in FL BFU-Es (~40%) than in BM BFU-Es (~15%), indicating an active cell cycle of FL BFU-E cells. Given that glucocorticoid enhances the self-renewal of BFU-E cells [9,18,19,20], that glucocorticoid receptor regulates *Myc* expression [9,21] and that *Myc* regulates the expression of *Ccna2* [22,23,24], our findings suggest that the *Nr3c1*-*Myc*-*Ccna2* axis contributes to the higher proliferation capability of FL BFU-E cells by enhancing the cell cycle.

### 3.4. GO Terms Involved in Heme/Hemoglobin Biosynthesis Were Upregulated in FL CFU-Es Compared to BM CFU-Es

**Figure 4:**
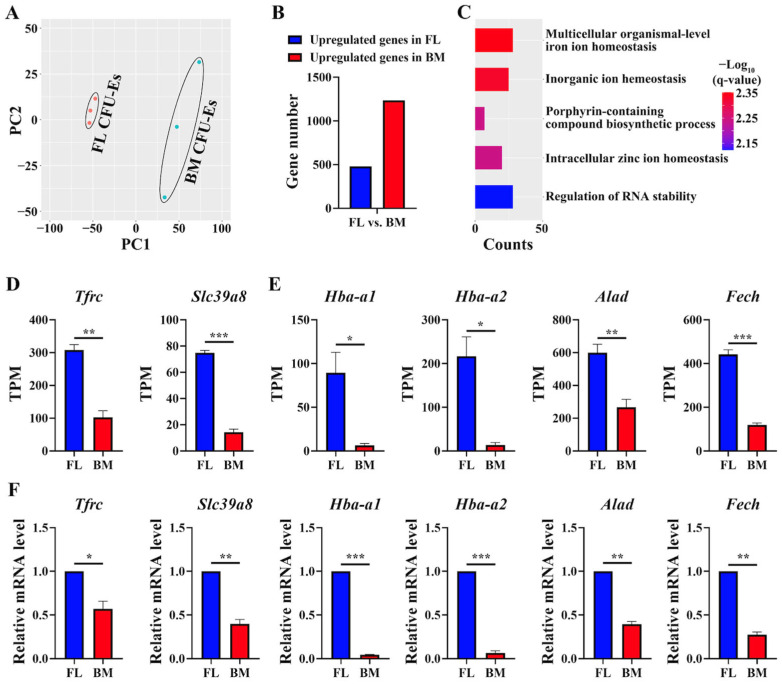
Comparisons between mouse FL and BM CFU-E cells. (**A**) Principal component analyses showing separation of FL CFU-Es and BM CFU-Es. (**B**) Bar plot of DEG numbers between FL CFU-Es and BM CFU-Es. (**C**) Bar plot of GO terms enriched in genes with higher expression in FL CFU-Es than BM CFU-Es. (**D**) Expression levels of *Tfrc* and *Slc39a8* in FL and BM CFU-Es from RNA-seq by TPM. (**E**) Expression levels of genes involved in hemoglobin biosynthesis in FL and BM CFU-Es from RNA-seq by TPM. (**F**) Expression levels of genes in D and E in FL and BM CFU-E cells as assessed by real-time PCR. *Gapdh* was used as control. N = 3. * *p* < 0.05. ** *p* < 0.01, *** *p* < 0.001. q-value represents adjusted *p*.

Next, we compared the transcriptomes of FL CFU-Es and BM CFU-Es. As shown in Figure 4A, PCA analysis revealed clear separation of FL CFU-Es and BM CFU-Es; both expressed around 9960 genes, among which 1073 genes were differentially expressed with 320 genes upregulated and 753 genes downregulated in FL CFU-Es compared to BM CFU-Es (Figure 4B). The DEGs are listed in Table S3. Analyses of the DEGs revealed that the top upregulated GO terms in FL CFU-Es included inorganic ion homeostasis and the porphyrin-containing compound biosynthetic process (Figure 4C). Specifically, the expression levels of transporters such as *Tfrc* (transferrin transporter) and *Slc39a8* (zinc importer), which was implicated in hemoglobin biosynthesis [25,26], were significantly higher in FL CFU-Es than in BM CFU-Es (Figure 4D). Moreover, the expression levels of hemoglobin genes *Hba-a1* and *Hba-a2* and gene-encoding enzymes involved in heme biosynthesis such as *Alad* and *Fech* were also significantly higher in FL CFU-Es compared to those in BM CFU-Es (Figure 4E). The differences in the expression of these genes were confirmed by real-time PCR (Figure 4F). Together, these findings suggest increased hemoglobin biosynthesis in FL CFU-Es and in BM CFU-Es.

### 3.5. GO Terms Involved in Immune Response/Immunoregulation Were Upregulated in BM Progenitors Than in FL Progenitors

**Figure 5:**
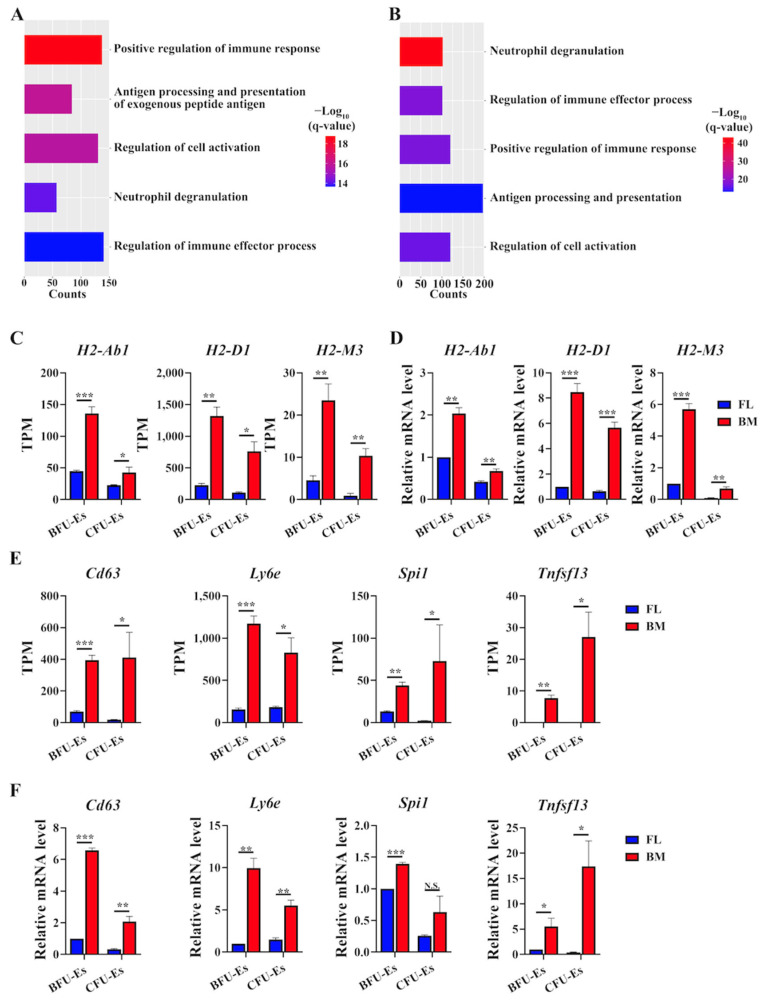
Upregulated GO terms and genes in BM progenitors than FL progenitors. (**A**) Bar plot of GO terms enriched in genes with higher expression in BM BFU-Es than FL BFU-Es. (**B**) Bar plot of GO terms enriched in genes with higher expression in BM CFU-Es than FL CFU-Es. (**C**) Expression level of histocompatibility complex genes from RNA-seq by TPM in FL and BM progenitors. (**D**) Expression level of histocompatibility complex genes as assessed by real-time PCR. (**E**) Expression level of marker genes involved in immunomodulation from RNA-seq by TPM in FL and BM progenitors. (**F**) Expression level of marker genes involved in immunomodulation as assessed by real-time PCR. *Gapdh* was used as control. N = 3. N.S.: Not Significant. * *p* < 0.05. ** *p* < 0.01, *** *p* < 0.001. q-value represents adjusted *p*.

We are also interested in genes with increased expression in BM progenitors compared to FL progenitors. Enriched GO terms of DEGs in both BFU-Es (Figure 5A) and CFU-Es (Figure 5B) included the upregulation of GO terms involved in the regulation of the immune response and antigen processing/presentation in BM erythroid progenitors compared to FL erythroid progenitors. A recent study identified an immune-prone erythroid cluster in human erythroblasts from the yolk sac, FL and BM by single-cell RNA-seq, and gene expression analyses of this cluster also showed GO enrichment in antigen processing/presentation [27]. Notably, the expression levels of a few major histocompatibility complex genes such as *H2-Ab1*, *H2-D1* and *H2-M3* were significantly higher in BM BFU-E and CFU-E than in their FL counterparts (Figure 5C). We further checked other marker genes of the immune-prone erythroid cluster such as *Cd63*, *Ly6e*, *Spi1* and *Tnfsf13* [27] and found higher expression levels of these genes in BM progenitors than in their FL counterparts (Figure 5E). The differences in the expression of these genes were confirmed by real-time PCR (Figure 5D,F). Our findings suggest that BM erythroid progenitors possess a higher immunomodulatory capability than FL erythroid progenitors.

### 3.6. Overall Conserved Temporal Patterns in Gene Expression during FL and BM Terminal Erythropoiesis

Next, we examined the similarities and differences between FL and BM definitive terminal erythropoiesis. PCA analyses revealed a clear separation of erythroblasts at each stage in both the FL (Figure S3A) and BM (Figure S3B). Distance analyses revealed similar temporal expression patterns during both FL (Figure S3C) and BM (Figure S3D) terminal erythropoiesis. The bar plot representation of the gene numbers expressed at each stage exhibited decreasing patterns during both FL and BM terminal erythropoiesis (Figure S3E). We further identified DEGs between adjacent stages. Notably, during both FL and BM terminal erythropoiesis, the most DEGs were found between Pros and Basos, followed by those found between Polys and Orthos, with very few DEGs between Basos and Polys (Figure S3F,G). These findings indicate overall conserved temporal patterns in gene expression between FL and BM terminal erythropoiesis.

### 3.7. Cluster Analyses Suggest Decrease in Protein Synthesis and Increase in Autophagy during Both FL and BM Terminal Erythropoiesis

**Figure 6:**
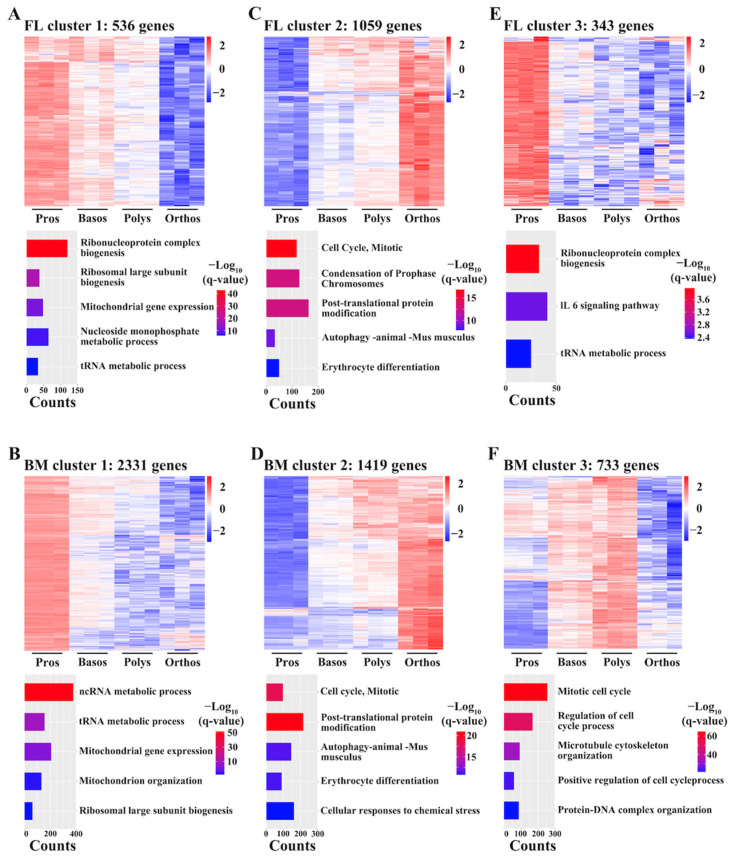
Clusters of DEGs between adjacent stages in FL and BM terminal erythropoiesis. Heatmap of gene expression and bar plot of enriched GO terms of genes in cluster 1 (**A**), cluster 2 (**C**) and cluster 3 (**E**) in FL terminal erythropoiesis. Heatmap of gene expression and bar plot of enriched GO terms of genes in cluster 1 (**B**), cluster 2 (**D**) and cluster 3 (**F**) in BM terminal erythropoiesis. q-value represents adjusted *p*.

We performed cluster analyses on the DEGs obtained from adjacent stage comparisons of the FL and BM separately. The DEGs in both the FL and BM were clustered into three groups. Genes in cluster 1 in both the FL (Figure 6A) and BM (Figure 6B) exhibited decreasing expression patterns and were enriched in the ncRNA metabolic process, ribosomal biogenesis, and tRNA metabolic process. In contrast, genes in cluster 2 in both the FL (Figure 6C) and BM (Figure 6D) exhibited increasing expression patterns and were enriched in post-translational protein modification and autophagy. Together, these results indicate a decrease in protein synthesis and an increase in protein and organelle clearance during both FL and BM terminal erythroid differentiation. Genes in cluster 3 were different between the FL and BM. The cluster 3 genes in the FL exhibited the highest expression of Pros and low expression of Basos, Polys and Orthos, and these genes were involved in ribonucleoprotein complex biogenesis (Figure 6E). Genes in cluster 3 in the BM had higher expression of Basos and Polys than Pros and Orthos, and these genes were involved in the cell cycle (Figure 6F). The differences of cluster 3 imply there are cell cycle differences between FL and BM terminal erythropoiesis.

### 3.8. Upregulation of GO Terms Involved in Translation and TCA Cycle in FL Erythroblasts and Upregulation of GO Terms Involved in Response to Virus in BM Erythroblasts

**Figure 7:**
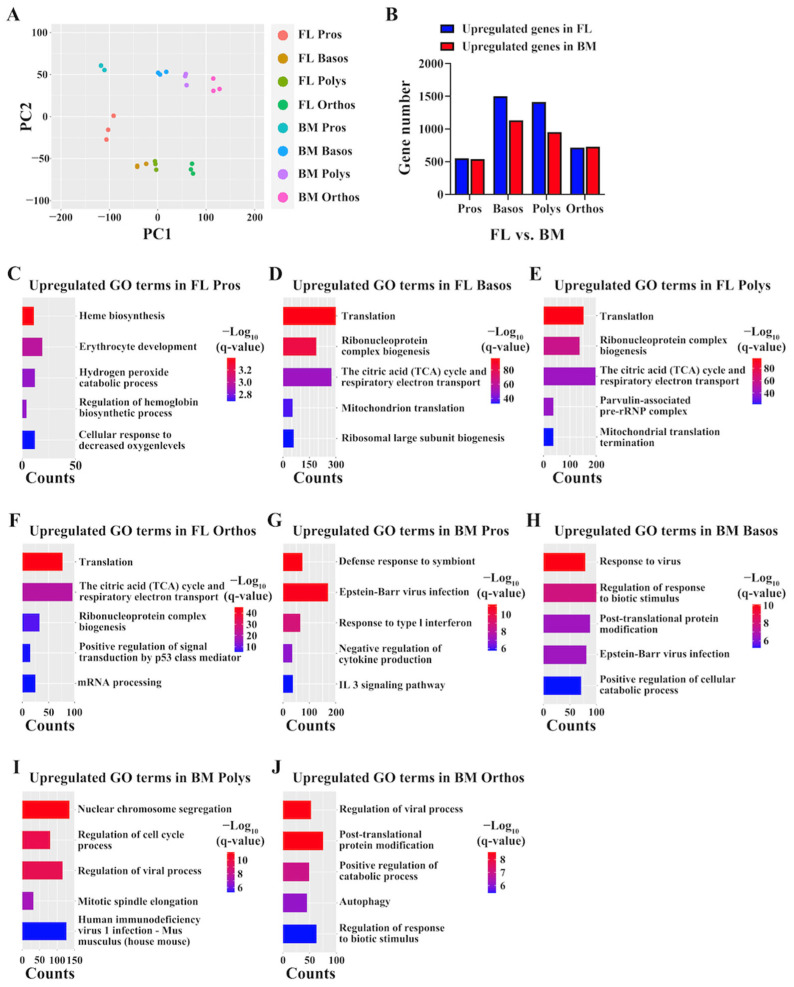
Same-stage transcriptome comparison between FL and BM erythroblasts. (**A**) Principal component analyses showing separation of FL and BM erythroblasts at all maturational stages. (**B**) Bar plot of numbers of DEGs at each stage between FL and BM erythroblasts. (**C**) Enriched GO terms of DEGs with increased expression in FL Pros compared to BM Pros. (**D**) Enriched GO terms of DEGs with increased expression in FL Basos compared to BM Basos. (**E**) Enriched GO terms of DEGs with increased expression in FL Polys compared to BM Polys. (**F**) Enriched GO terms of DEGs with increased expression in FL Orthos compared to BM Orthos. (**G**) Enriched GO terms of DEGs with increased expression in BM Pros compared to FL Pros. (**H**) Enriched GO terms of DEGs with increased expression in BM Basos compared to FL Basos. (**I**) Enriched GO terms of DEGs with increased expression in BM Polys compared to FL Polys. (**J**) Enriched GO terms of DEGs with increased expression in BM Orthos compared to FL Orthos. q-value represents adjusted *p*.

Next, we performed the same stage comparison between FL and BM erythroblasts. PCA revealed a clear separation of samples not only between different maturational stages but also within the same stage between the FL and BM (Figure 7A), indicating differences between FL and BM erythroblasts. Indeed, 704, 1918, 1509 and 784 DEGs were identified between Pros, Basos, Polys and Orthos of the FL and BM. Among these DEGs, 277, 1113, 831 and 358 DEGs showed higher expression in the FL, while 427, 804, 678 and 426 DEGs showed higher expression in the BM (Figure 7B). The lists of DEGs at each stage are provided in Table S4. The GO terms of DEGs with increased expression in FL Pros, Basos, Polys and Orthos compared to their BM counterparts are shown in Figure 7C–F, respectively. As shown in Figure 7C, similar to the difference at the CFU-E stage, the top upregulated GO terms in FL Pros compared to BM Pros included heme/hemoglobin biosynthesis. Notably, the GO terms for translation and TCA cycle were upregulated in FL Basos (Figure 7D), Polys (Figure 7E) and Orthos (Figure 7F) compared to their BM counterparts, which may contribute to the larger cell size of FL erythroblasts. In contrast, the GO term for the response to viruses was upregulated in BM erythroblasts at all maturational stages compared to their FL counterparts (Figure 7G–J), indicating the exposure of erythroblasts to viruses in the BM but not in the FL.

### 3.9. Specifically Expressed Genes in FL or BM Erythroblasts

**Figure 8:**
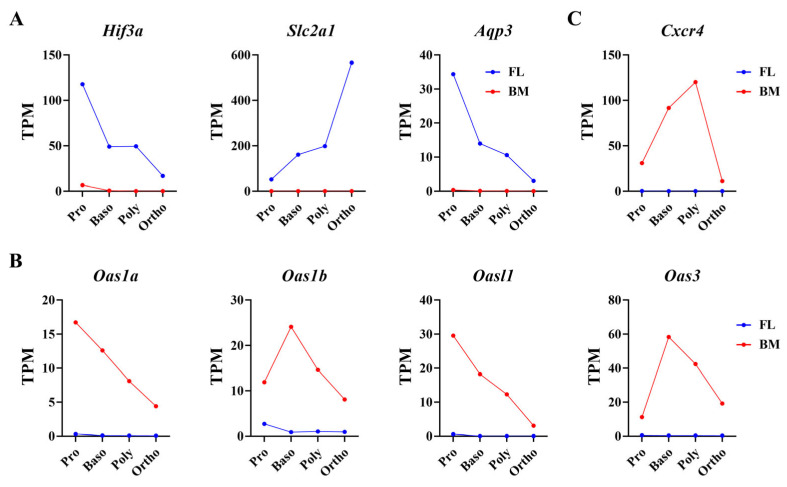
Specifically expressed genes in FL or BM erythroblasts. (**A**) Bar plot of expression of hypoxia-regulated genes *Hif3a*, *Aqp3* and *Slc2a1* from RNA-seq by TPM. (**B**) Bar plot of expression of genes involved in antiviral signaling pathway from RNA-seq by TPM. (**C**) Bar plot of Cxcr4 expression from RNA-seq by TPM. q-value represents adjusted *p*.

To further investigate the differences between FL and BM terminal erythropoiesis, we sought to identify genes with drastically different expression levels (minimum eight-fold) between the FL and BM across all terminal maturational stages and found a total of 116 such genes. The list of these genes is provided in Table S5. Among these genes, 43 genes were significantly more highly expressed in FL erythroblasts at all maturational stages compared to their BM counterparts. Notably, hypoxia-regulated genes such as *Hif3a, Slc2a1* (encoding Glut1) and *Aqp3* were only expressed in FL erythroblasts (Figure 8A). On the other hand, the expression levels of 73 genes were drastically higher in BM erythroblasts than their FL counterparts. Specifically, the family of 2′-5′-oligoadenylate synthetases (OASs) *Oas1a*, *Oas1b, Oas3 and Oasl1*, interferon-induced enzymes which are all involved in the antiviral signaling pathway [28], were only expressed in BM erythroblasts (Figure 8B), indicating the exposure of BM erythroblasts to viruses. Additionally, *Cxcr4*, the receptor for chemokine *Cxcl12* [29], was also specifically expressed in the BM (Figure 8C). Consistent with the RNA-seq results, we could not detect the expression of *Hif3a, Slc2a1* and *Aqp3* in BM erythroblasts although we detected their expression in FL erythroblasts by real-time PCR. Similarly, we could not detect the expression of *Oas1a*, *Oas1b, Oas3, Oasl1* and *Cxcr4* in FL erythroblasts although we were not able to detect their expression in FL erythroblasts by real-time PCR. Together, these findings indicate different microenvironments for FL and BM erythropoiesis.

## 4. Discussion

The present study presents a comprehensive transcriptomic and characteristics comparison of FL and BM definitive erythroid cells at all maturational stages from BFU-Es to orthochromatic erythroblasts. These comparative studies have uncovered previously unrecognized characteristics of murine FL and BM definitive erythroid cells as well as their similarities and differences, providing novel insights into erythrocyte biology. The transcriptomes of erythroid cells at all maturational stages lay the foundation for future mechanistic studies.

It has been known for a long time that red blood cells generated by FL definitive erythropoiesis are macrocytic and hyperchromatic. Indeed, we have recently shown that the mean corpuscular volume of red blood cells from neonatal mice is ~2 times larger than that of red blood cells from adult mice [30]. However, the underlying mechanisms for the larger FL red blood cells remain unclear. Based on the findings that red blood cell size is negatively correlated with the number of erythroblast cell division [31,32,33,34], it was thought that the larger size of FL-generated red blood cells could be attributed to the skipping of a cell division during the terminal erythroid differentiation of FL definitive erythropoiesis. Here we found that FL erythroid cells at all maturational stages were larger than their BM counterparts. We also showed that FL and BM CFU-Es underwent similar numbers of cell division. Our findings strongly suggest that the larger cell size of FL-generated red blood cells is not due to a decreased cell division number of FL erythroblasts. A recent study showed that EPO/EPOR signaling increased red blood cell size while also paradoxically increasing the number and speed of erythroblast cell cycles [35]. However, it should be noted that EPO/EPOR signaling only increased the red blood cell size by ~20%; thus, increased blood EPO levels in fetuses or neonates could not explain the two-times differences in cell size between the FL-generated and BM-generated red blood cells [30,36,37]. Interestingly, our transcriptome comparisons revealed that translation and TCA were significantly more upregulated in FL erythroblasts compared to BM erythroblasts, likely due to the need for more protein synthesis and energy production for the larger cells [38,39,40]. Together, these findings indicate that larger cell size is an intrinsic characteristic of FL erythroid cells starting from the earliest committed erythroid progenitors.

In contrast to the extensive studies on mouse FL erythroid progenitor BFU-E and CFU-E cells [9,18,41,42], our knowledge on BM erythroid progenitors is very limited due to the lack of methods to isolate mouse BM erythroid progenitors. We recently developed a method to purify mouse BM BFU-E and CFU-E cells [8] which enabled us not only to obtain the transcriptomes of BM BFU-E and CUF-E cells but also to compare them with their FL counterparts. One interesting finding is that FL BFU-E cells exhibit higher proliferation capacity and express higher levels of genes involved in the cell cycle than BM BFU-E cells. Specifically, FL BFU-E cells express higher levels of glucocorticoid receptor *Nr3c1*, *Myc* and *Ccna2*. It has been reported that dexamethasone treatment led to increased expression of Myc in FL BFU-E cells [9]. It also has been reported that Myc serves as the transcription factor for Ccna2 [22,23,24]. These findings indicate the role of the Nr3c1-Myc-Ccna2 axis in the hyperproliferation of FL BFU-E cells and provide novel insights into the mechanisms of the effect of glucocorticoid on BFU-E cells. Another major difference between FL and BM BFU-E cells is that while the proliferation capacity of FL BFU cells is highly heterogeneous, that of BM BFU-E cells is relatively homogenous. In future studies, it will be important to investigate the molecular basis for the heterogeneity of FL BFU-E cells and define the genes which are responsible for the highly proliferative capacity of FL BFU-E cells using a single-cell RNA-seq approach. The results from such studies should have implications in developing strategies to enhance ex vivo red blood cell production. The third difference is that FL BFU-Es underwent two more cell divisions compared to BM BFU-E cells. Interestingly, the genes involved in telomere maintenance, such as Ccne1, Ccne2, Rad51 and Dna2 (Table S2), exhibited higher expression in FL BFU-Es than BM BFU-Es. Given the important role of telomeres in cell division [43,44,45], it is reasonable to speculate a potential role of enhanced telomere maintenance in the cell division of FL BFU-E cells.

At the CFU-E stage, one of the major differences between the FL and BM is that GO terms involved in ion homeostasis and heme biosynthesis are highly enriched in FL CFU-Es. Interestingly, in addition to the upregulation of transferrin receptor *Tfrc*, the expression of a zinc importer *Slc39a8* is also significantly higher in FL CFU-Es compared to BM CFU-Es. Together with the previous findings that cellular zinc deficiency impairs heme biosynthesis [25,46] and that zinc deficiency has been associated with anemia in humans [47,48], our findings suggest the potentially important roles of the zinc transporter in hemoglobin biosynthesis or/and erythropoiesis which is currently understudied and warrants further investigation.

Red blood cells are traditionally thought of as only inert oxygen carriers. Interestingly, there is increasing evidence that CD71^+^-nucleated erythroid cells possess an immunomodulatory function [27,49,50,51]. A recent study identified a subset of immunomodulatory human erythroblasts present from the yolk sac to the adult BM throughout human ontogenesis [27]. But it is unclear whether erythroid progenitors also possess such a function. Our findings that both BFU-Es and CFU-Es express immunomodulatory genes strongly suggest that the immunomodulatory function of erythroid cells starts from the earliest-committed-stage BFU-Es. Moreover, our findings that GO terms enriched in immunoregulation are significantly higher in BM erythroid progenitors indicate a stronger immunomodulatory capacity of BM erythroid progenitors, reflecting the more complex responses in the BM than in the FL.

We also compared the transcriptomes of erythroblasts at all maturational stages between the FL and BM and found overall conserved temporal patterns. Consistent with previous RNA-seq analyses on human erythroblasts [52], gene numbers expressed at each stage decreased during both FL and BM definitive terminal erythropoiesis, indicating that the downregulation of transcription is a conserved feature between human and mouse definitive terminal erythropoiesis. Notably, while more than 1000 genes were differentially expressed between Pros and Basos and between Polys and Orthos in both the FL and BM, very few DEGs were found between Basos and Polys. The same change patterns were also found in primary human FL erythroblasts [53] but not in in-vitro-cultured erythroblasts derived from CD34^+^ cells from human cord blood [52], peripheral blood [54] and the FL [53]. These findings suggest that dramatic changes occur during the transition from Pros to Basos and from Polys to Orthos (likely to prepare for enucleation) but not from Basos to Polys in vivo.

Another interesting finding is the exclusive expression of certain genes in either FL or BM erythroblasts. Our finding that Glut1 was only expressed in FL erythroblasts is consistent with a previous report that Glut1 was transiently expressed in erythrocytes during the neonatal period in mice [55,56]. Interestingly, out of the three hypoxia-inducible factors Hif1a, Hif2a and Hif3a, Hif3a is the only family member differentially expressed between FL and BM erythroblasts, suggesting the role of Hif3a in the response of FL erythroblasts to hypoxia. In the case of Aqp3, a previous comparative study of FL primitive and BM definitive erythroid precursors reported that *Aqp3* is only expressed in FL primitive erythroblasts and can accumulate significant amounts of exogenous H_2_O_2_ [6]. Here, we found that *Aqp3* is also abundantly expressed in FL but not BM definitive erythroblasts, indicating *Aqp3* is a marker of fetal liver erythropoiesis. In marked contrast, members of the OAS family, which are interferon-induced genes and play important antiviral roles [57,58,59], were detected in BM erythroblasts but not expressed FL erythroblasts at all. These findings are in line with the facts that the fetus is protected from viral infection by the placenta [60,61]. Another gene that was only expressed in BM erythroblasts is Cxcl12 receptor *Cxcr4*. Given the critical role of CXCR4–CXCL12 interaction in the homing/retention of hematopoietic stem and progenitor cells [62,63], our findings suggest a mechanism for the retention of erythroblasts in the BM, while such a mechanism likely does not exist in the FL.

## Data Availability

The original data presented in the study are openly available in GEO at GSE269127.

## Supplementary Materials

The following supporting information can be downloaded at: https://www.mdpi.com/article/10.3390/cells13131149/s1, Figure S1: Gating procedure and isolation of FL and BM progenitors and erythroblasts; Figure S2: Cell division of FL and BM progenitor cells; Figure S3: Temporal patterns in gene expression in FL and BM terminal erythropoiesis; Table S1: Reagents and primers; Table S2: DEGs between FL BFU-Es and BM BFU-Es; Table S3: DEGs between FL CFU-Es and BM CFU-Es; Table S4: DEGs from same-stage comparison between FL and BM erythroblasts; Table S5: Specifically expressed genes in FL or BM erythroblasts.
