# Supplementary material for: Global Transcriptomic and Characteristics Comparisons between Mouse Fetal Liver and Bone Marrow Definitive Erythropoiesis
Source: Cells. 2024 Jul 5;13(13):1149. doi: 10.3390/cells13131149 (PMC11240549; doi:10.3390/cells13131149)
Supplement: Supplementary file 1 [file cells-13-01149-s001.zip › Supplementary materials_052824.pdf]

SUPPLEMENTARY FIGURES

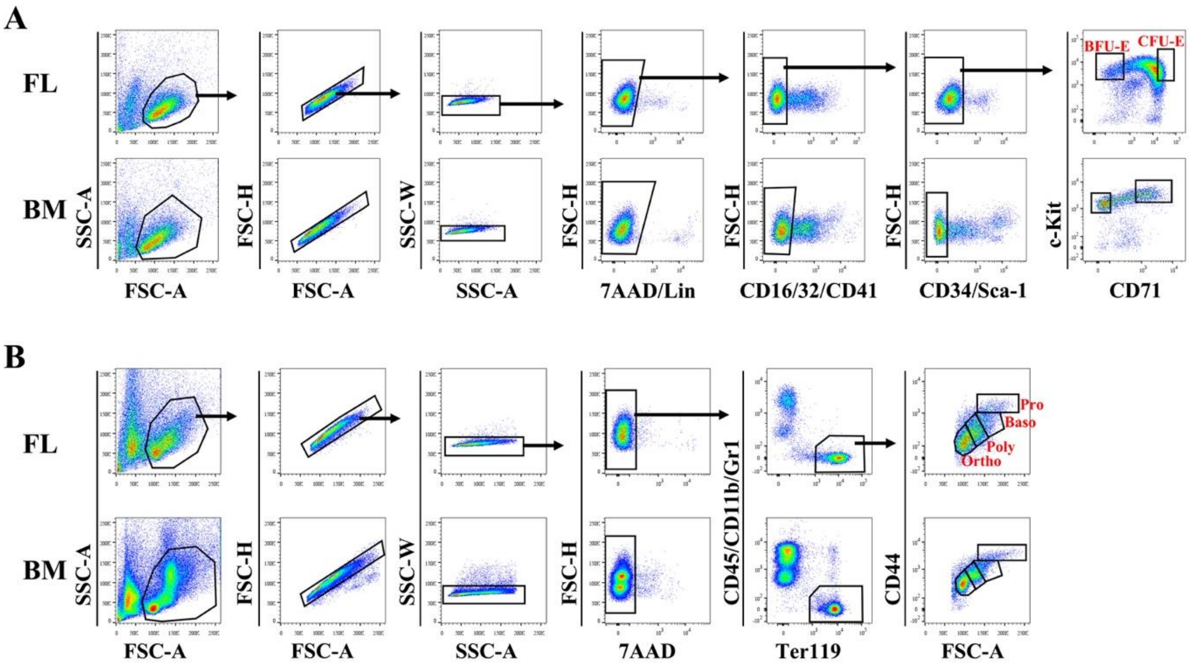

Figure S1. Gating procedure and isolation of FL and BM progenitors and erythroblasts. **(A)** Gating strategy for FL and BM BFU-E (cKit^{+}CD71^{-}) and CFU-E (cKit^{+}CD71^{high}). **(B)** Gating strategy for FL and BM erythroblasts (plot of CD44 versus FSC-A with gating the population proerythroblasts (Pro), basophilic (Baso), polychromatic (Poly), orthochromatic (Ortho) erythroblasts).

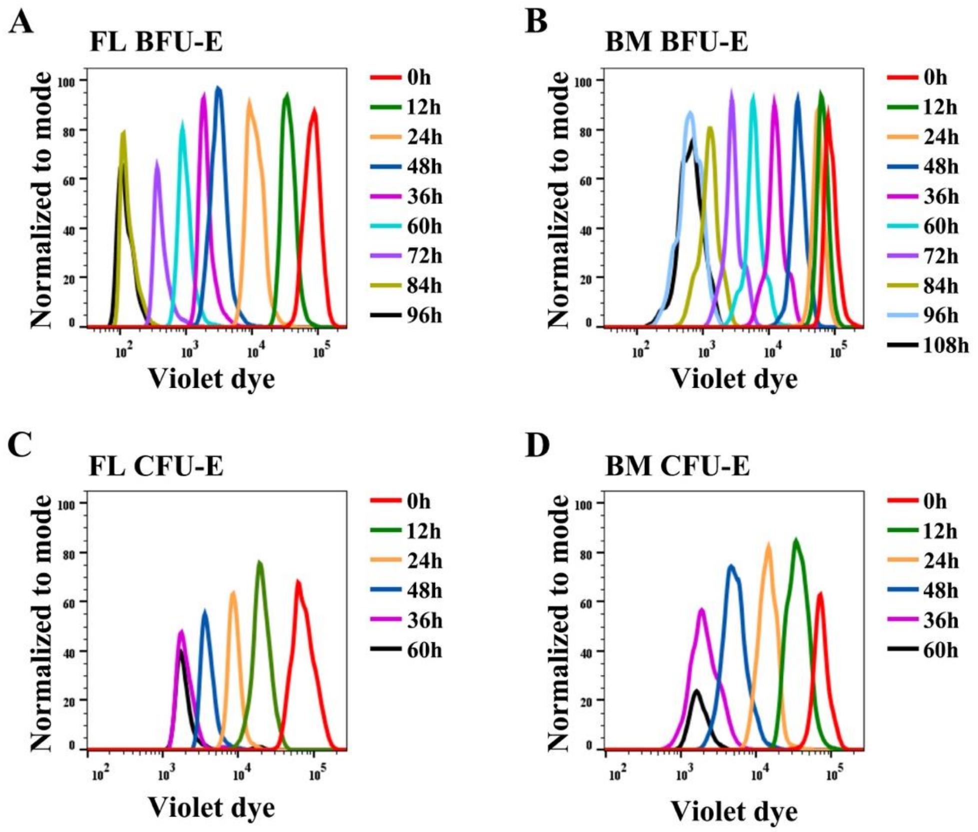

Figure S2. Cell division of FL and BM progenitor cells. (A) An overlay of the Violet Cell Trace signals every 12hrs from 0h to 96h of FL BFU-E cultured in vitro. **(B)** An overlay of the Violet Cell Trace signals every 12hrs from 0h to 108h of BM BFU-E cultured in vitro. **(C)** An overlay of the Violet Cell Trace signals every 12hrs from 0h to 60h of FL CFU-E cultured in vitro. **(D)** An overlay of the Violet Cell Trace signals every 12hrs from 0h to 60h of BM CFU-E cultured in vitro.

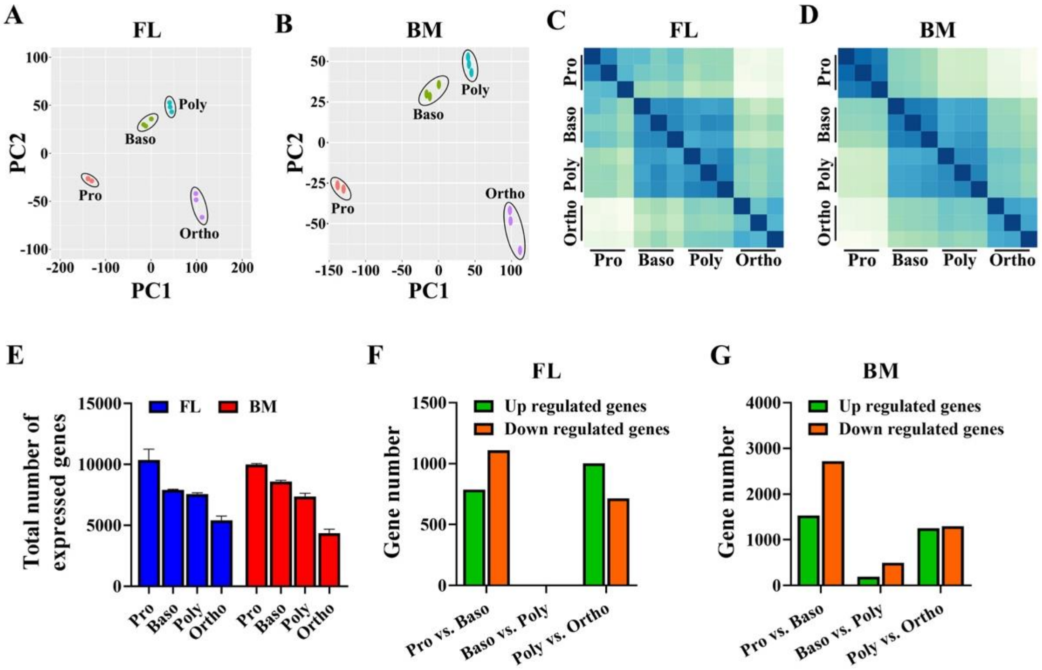

Figure S3. Temporal patterns in gene expression in FL and BM terminal erythropoiesis. **(A)** Principal component analyses showing the separation of stages in FL terminal erythropoiesis. **(B)** Principal component analyses showing the separation of stages in BM terminal erythropoiesis. **(C)** Heatmap of distances among different stages showing strong similarity of Baso and Poly in FL terminal erythropoiesis. **(D)** Heatmap of distances among different stages showing strong similarity of Baso and Poly in BM terminal erythropoiesis. **(E)** Bar plot of numbers of expressed genes at each stage in FL and BM terminal erythropoiesis. **(F)** Bar plot of numbers of DEGs between adjacent stages in FL terminal erythroblasts. **(G)** Bar plot of numbers of DEGs between adjacent stages in BM terminal erythroblasts.

SUPPLEMENTARY TABLES

Table S1 **Reagents and primers**

Table S2 **DEGs between FL BFU-E and BM BFU-E**

Table S3 **DEGs between FL CFU-E and BM CFU-E**

Table S4 **DEGs from same-stage comparison between FL and BM erythroblasts**

Table S5 **Specifically expressed genes in FL or BM erythroblasts**
